# Genome-Wide Identification of Small RNAs in *Bifidobacterium animalis* subsp. *lactis* KLDS 2.0603 and Their Regulation Role in the Adaption to Gastrointestinal Environment

**DOI:** 10.1371/journal.pone.0117373

**Published:** 2015-02-23

**Authors:** De-Quan Zhu, Fei Liu, Yu Sun, Li-Mei Yang, Li Xin, Xiang-Chen Meng

**Affiliations:** 1 Key Laboratory of Dairy Science, Ministry of Education, Northeast Agricultural University, Harbin, People’s Republic of China; 2 Synergetic Innovation Center of Food Safety and Nutrition, Northeast Agricultural University, Harbin, People’s Republic of China; 3 College of Life Science, Jiamusi University, Jiamusi, People’s Republic of China; Cornell University, UNITED STATES

## Abstract

**Objective:**

*Bifidobacteria* are one of the predominant bacterial species in the human gastrointestinal tract (GIT) and play a vital role in the host’s health by acting as probiotics. However, how they regulate themselves to adapt to GIT of their host remains unknown.

**Methods:**

Eighteen *bifidobacterial* strains were used to analyze their adaptive capacities towards simulated GIT environment. The strain with highest survival rate and adhesion ability was selected for comparative genome as well as transcriptomic analysis.

**Results:**

The *Bifidobacterium animalis* subsp. *lactis* KLDS 2.0603 strain was demonstrated to have the highest survival rate and adhesion ability in simulated GIT treatments. The comparative genome analysis revealed that the KLDS 2.0603 has most similar whole genome sequence compared with BB-12 strain. Eleven intergenic sRNAs were identified after genomes prediction and transcriptomic analysis of KLDS 2.0603. Transcriptomic analysis also showed that genes (mainly sRNAs targeted genes) and sRNAs were differentially expressed in different stress conditions, suggesting that sRNAs might play a crucial role in regulating genes involved in the stress resistance of this strain towards environmental changes.

**Conclusions:**

This study first provided deep and comprehensive insights into the regulation of KLDS 2.0603 strain at transcription and post-transcription level towards environmental.

## Introduction

*Bifidobacteria* are predominant bacterial species in the human and animal gastrointestinal tracts (GIT) [1] and they have a beneficial effect on host’s health by regulating intestinal microbial homeostasis, inhibiting pathogens and harmful bacteria, improving the gut mucosal barrier and decreasing lipopolysaccharide levels in the intestine [2, 3]. The genus *Bifidobacterium* consisted of 37 species, including *B. animalis* (containing *B. animalis* subsp. *animalis* and *lactis*), *B. bifidum, B. pseudolongum* (containing *B. pseudolongum* subsp. *globosum* and *pseudolongum*), *B. longum* (containing *B. longum* subsp. *infantis, longum* and *suis*), etc [4]. Some *Bifidobacterium* strains are considered as important probiotics and often used in the food industry due to the ability to survive and colonize in the host’s intestinal tract. However, most *Bifidobacteria* are sensitive to the stresses (e.g. heat, cold, exposure to oxygen, acid, bile, osmotic condition, etc), which usually affect their viability and reduce their probiotic effects [5, 6]. Thus, it is necessary to select *Bifidobacteria* isolates with high survival rate and high adhesion ability in GIT environment and explore their underlying molecular mechanism.

Bacterial small RNAs (sRNAs) are known to be the key regulators and they regulate gene expression through base-pairing with downstream target mRNAs to attenuate translation of mRNA into protein at the post-transcriptional level [7]. These molecules are involved in a variety of cellular processes including stress responses to quorum sensing [8], pathogenesis and virulence [9], developmental processes [10] and varying environment [11–13]. At present, genomewide analysis to identify sRNAs has been largely based on microarrays and next-generation sequencing technologies. The RNA sequencing technology, in particular, has revolutionized sRNA discovery by transcriptomic data. The deep sequencing has emerged as a powerful experimental method for transcriptome analysis and detecting sRNAs in a wide variety of microbial genomes, including those of *Escherichia coli* [14], *Bacillus subtilis* [15], *Mycobacterium tuberculosis* [16], *Helicobacter pylori* [17], *Streptomyces coelicolor* [18], *Salmonella enteric* [19], *Vibrio cholerae* [20], *Listeria monocytogenes* [10] and *Streptococcus pneumoniae* [21].

Transcriptional regulatory programs and sRNA contain the information to allow the bacteria to adapt to different situations. Although *bifidobacteria* are widely used as food supplements for their probiotic effect, genome-wide characterization of sRNAs under different environmental stress and their role in regulatory networks are still not well understood. In our study, *Bifidobacterium animalis* subsp. *lactis* KLDS 2.0603 with high survival and adhesion ability was isolated in our lab from 18 *bifidobacterial* strains kept by KLDS-DICC. The whole transcriptome sequences of KLDS 2.0603 were performed based on comparative genome analysis of this strain and other 9 reference strains. We also first reported the genomewide analysis of sRNAs and genes regulated by these sRNAs involved in the resistance to environmental stress (simulated GIT conditions) in KLDS 2.0603.

## Materials and Methods

### Bacterial strains and culture conditions

The 18 *bifidobacterial* strains and other three reference strains used in this study (**Table 1 in S1 File**) were provided by our lab (Key Lab of Dairy Science [KLDS], Ministry of Education, Northest Agricultural University, China). *Bifidobacterial* strains were cultured on modified De Man Rogosa Sharpe (mMRS) medium (Oxoid, Hampshire, UK) supplemented with 0.05% (w/v) L-cysteine hydrochloride under anaerobic conditions at 37°C. For anaerobic cultivation, an anaerobic incubator (Thermo Fisher Scientific, Fisher Canada, Nepean, ON, Canada) was used. *Lactobacillus rhamnosus* and *Lactobacillus acidophilus* were cultivated overnight in MRS broth (Oxoid, Hampshire, UK) at 37°C under aerobic condition.

### Caco-2 cell line and culture condition

The Human colon adenocarcinoma (Caco-2) cell line obtained from China Cell Bank (Shanghai, China) was utilized for the cell adhesion experiments of *bifidobacterial* and reference strains. The cells were grown in Dulbecco’s modified Eagle’s Medium (DMEM; Gibco, Grand Island, NY) supplemented with 10% heat-inactivated (56°C, 30 min) fetal bovine serum (Gibco, Grand Island, NY) and 1% antibiotics at 37°C, 5% CO_2_.

### Survival of *bifidobacteria* in simulated GIT conditions

**Artificial saliva** was prepared by dissolving 6.2 g NaCl, 2.2 g KCl, 0.22 g CaCl_2_, and 1.2 g NaHCO_3_ in 1 L of distilled water [22]. After sterilization by autoclaving, the solution was cooled to approximately 20°C, and 3.0 g/L α-amylase (Sigma, St Louis, MO, USA) was added followed by pH adjustment to 6.9. Then the saliva was filtered through a 0.22-μm filter (cellulose, Millipore Corp., Bedford, MA). **Simulated gastric fluid** was prepared according to the method of Khalf *et al*. [23] using 1 L solution of 3.0 g NaCl, 1.1 g KCl, 0.15 g CaCl_2_, and 0.60 g NaHCO_3_. The solution was sterilized by autoclaving, and cooled to approximately 20°C, followed by addition of 3.0 g/L pepsin from porcine stomach mucosa (Sigma, St Louis, MO, USA) and pH adjustment to 2 with 10 N HCl. Then the gastric fluid was filtered through a 0.22-μm filter (cellulose, Millipore Corp., Bedford, MA). **Artificial small intestinal fluid** was prepared by dissolving 5.0 g NaCl, 0.60 g KCl, 0.30 g CaCl_2_, and 0.60 g NaHCO_3_ in 1 L of distilled water. The solution was sterilized by autoclaving, and cooled to approximately 20°C prior to use. After addition with 3.0 g/L OX gall (Sigma, St Louis, MO, USA), 1.0 g/L precreatin (Sigma, St Louis, MO, USA) and 3.0 g/L pepsin (Sigma, St Louis, MO, USA) [23] the intestinal fluid was filtered through a 0.22-μm filter (cellulose, Millipore Corp., Bedford, MA).

To determine the survival rates of *Bifidobacterial* and reference strains in simulated GIT conditions, bacteria cells passed sequentially through the in *vitro* GIT model developed by Weiss et al. [24]. Briefly, the model consisted of three compartments, including the mouth, stomach and small intestine. First, washed bacterial cells (5 mL, 1 × 10^9^ CFU/ml) were suspended in 5 mL saliva (pH 6.9) for 5 min. Then the samples were resuspended to 10 mL gastric fluid (total pH = 3.0) and incubated for 2 h. Finally, the samples were resuspended to 10 mL intestinal fluid with 1M NaHCO_3_ added (total pH = 7.6) and incubated for 2 h. The entire digestion procedure was performed at 37°C, with stirring at 50 rpm to simulate peristaltic contraction.

After treatment with simulated GIT fluid, the bacteria cell suspensions were diluted 10-fold and plated onto mMRS or MRS agar plates. The number of colonies was counted following 36 h of incubation at 37°C under anaerobic conditions or aerobic condition. The survival rate was calculated as follows:

Survival % = CFUml treatmentCFUml control × 100

### Adhesion analysis of *bifidobacterial* strains to Caco-2 cells by direct microscopic examination method

The Caco-2 cells were used for adhesion assays after 15–17 d of cultivation as described above and complete differentiation. The culture medium was replaced by an antibiotic-free medium one day prior to the adhesion assay. Cell monolayers were carefully washed twice with phosphate-buffered saline (PBS) before bacteria cells were added. Then the cells of treated *bifidobacterial* and reference strains were added into the prepared cell monolayers. After 2 h of incubation at 37°C, all monolayers were washed 5 times with PBS (pH = 7.2) to release unbound bacteria. Then the cells were fixed with methanol for 10 min, Gram stained and counted using 20 randomized microscopic fields per well. Adhesion activity was indicated as the number of bacteria adhering to 100 cells.

### Adhesion analysis of *bifidobacterial* strains to Caco-2 cells by plate count method

The treatments on Caco-2 cells and *bifidobacterial* strains were the same with direct microscopic examination method until 5 times washing with PBS (pH = 7.2) to release unbound bacteria. Then the cells were treated with 250 μL 0.25% trypsin and incubated at 37°C for 15 min to release the adherent bacteria. Finally, 250 μL DMEM/10% fetal bovine serum was added to terminate the activity of trypsin. The bound bacteria were diluted 10-fold and plated onto mMRS agar plates. The number of colonies was counted following 36 h of incubation at 37°C under anaerobic conditions or aerobic condition. Invasion assay was run in triplicate for each isolate, and invasion ability of bacteria was reflected as Log CFU/well.

### Stress sample preparation for transcriptomic analysis

The KLDS 2.0603 cells grown in mMRS broth were harvested by centrifugation, washed twice with PBS (pH = 7.2), and then resuspended in PBS (pH = 3.0) for 2 h (acid stress), 0.3% bile salts for 2 h (bile stress) and sequential simulated GIT fluids (saliva fluids for 5 min, gastric fluids for 2 h and intestinal fluids for 2 h), respectively. KLDS 2.0603 cells untreated was considered as control.

### RNA isolation

The differently treated and untreated *Bifidobacterium* cells were collected by centrifugating at 8 000 × g for 10 min and mixed with 2-fold volume of RNA protect reagent (Qiagen, Valencia, CA, USA). The pellets were re-suspended in Tris-EDTA (TE) buffer containing 15 mg/mL lysozyme (Sigma–Aldrich, China) and incubated at 37°C for 15 min. Then total RNA from each sample was extracted from using Trizol reagent (Invitrogen, Grand Island, NY) and purified with the RNA easy minikit (Qiagen, Valencia, CA, USA) following the manufacturer's instructions [25], followed by DNase I (Qiagen, Valencia, CA, USA) to remove genomic DNA contamination. Then total RNA quality and integrity was analyzed by agarose gel electrophoresis and the Agilent 2100 Bioanalyzer (Agilent Technologies, Böblingen, Germany) [26], all samples had an RNA Integrity Number (RIN) value above 8.

### Library construction and sequencing

After validating the integrity and purity of total RNA, cDNA library was constructed according to the protocol of TruSeq RNA Sample Prep Preparation Kit (RS-930-2001; Illumina, San Diego, CA). Briefly, the poly-A containing mRNA molecules were purified using poly-T oligo-attached magnetic beads (Illumina, San Diego, CA) from total RNA, and sheared to small fragments using divalent cations under elevated temperature. The cleaved RNA fragments were copied into first strand cDNA using reverse transcriptase and random primers, followed by second strand cDNA synthesis using DNA Polymerase I and RNase H. These cDNA fragments were subjected to end-repair using 3ʹ–>5ʹ exonuclease and polymerase, then go through the addition of a single ‘A’ base and subsequent adapter-ligation. The unsuitable fragments were removed using AMPureXP beads (Beckman Coulter Inc, Brae, CA, USA), and the sequencing library was constructed with PCR amplification. After quantified using the Quant-iT PicoGreen ds DNA Assay Kit (Invitrogen, Eugene, OR, USA) and fluorospectrophotometry and quantified with Agilent 2100 Bioanalyzer (Agilent Technologies, Böblingen, Germany), the multiplexed DNA libraries were mixed by equal volume with normalized 10 nM concentration. Finally, the sequencing library was then sequenced on Illumina Hiseq2000 platform (Shanghai Personal Biotechnology Cp., Ltd. Shanghai, China).

### Public data access and sequencing data production

The whole genome sequence analysis and the genome annotation of KLDS 2.0603 strain have been done in our lab. In addition, the genome sequences and the annotation files of the other 9 bacteria strains were downloaded from NCBI. The 9 reference strains included *Bifidobacterium animalis* subsp. *Animalis* ATCC 25527 and *lactis* BB-12, *Bifidobacterium breve* UCC2003, *Bifidobacterium longum* subsp. *longum* NCC2705 and *infantis* ATCC 15697, *Bifidobacterium adolescentis* ATCC 15703, *Bifidobacterium bifidum* PRL2010, *Lactobacillus rhamnosus* GG and *Lactobacillus acidophilus*. The genomic information of KLDS 2.0603 and other 9 reference strains was shown in **Table 1.**

**Table 1:** The genomic information of KLDS 2.0603 and other 9 reference strains.

Sample genome name	Nucleotide sequence database accession number	Length (bp)	Annotated gene number	tRNA number	tRNA synthetases number	rRNA operon number	Gene coding content	GC content
ATCC-15697	CP001095	2,832,748	2416	79	18	5(5S/16S/30S/23S/50S)	85.02%	59.86%
ATCC-15703	AP009256	2,089,645	1631	54	21	5(5S/16S/30S/23S/50S)	86.50%	59.18%
ATCC-25527	CP002567	1,932,693	1538	52	20	5(5S/16S/30S/23S/50S)	85.33%	60.47%
BB-12	CP001853	1,942,198	1642	52	17	3(5S/16S/23S)	89.86%	60.48%
Lactobacillus-acidophilus-NCFM	CP000033	1,993,560	1832	61	17	5(5S/16S/30S/23S/50S)	87.28%	34.71%
Lactobacillus-rhamnosus-GG	FM179322	3,010,111	2944	57	20	5(5S/16S/30S/23S/50S)	85.27%	46.69%
NCC2705	AE014295	2,256,640	1727	57	18	5(5S/16S/30S/23S/50S)	85.30%	60.12%
PRL2010	CP001840	2,214,656	1707	52	19	5(5S/16S/30S/23S/50S)	83.63%	62.66%
UCC2003	CP000303	2,422,684	1854	54	19	5(5S/16S/30S/23S/50S)	84.06%	58.73%
KLDS 2.0603	CP007423	1,946,899	1747	52	19	5(5S/16S/30S/23S/50S)	86.66%	60.48%

Furthermore, the transcriptomic data of KLDS 2.0603 strain under different GIT conditions were also collected and deposited in NCBI BioProject under PRJNA252998 accession number, with BioSample accession number of SAMN03072820, experimental number of SRX703605, and RUN number of SRR1577882.

### Selection of intergenic regions (IGR)

In order to predict the sRNA information of KLDS 2.0603 and other 9 reference strains, the Bio::SeqIO package (http://doc.bioperl.org/releases/bioperl-1.0.1/Bio/SeqIO.html) of Perl was utilized to select IGR sequences according to the genome annotation information [27]. In order to remove the possible promoters, untranslated region, as well as terminators of the annotated open reading frames (ORF), 40 bp on both sides of the original IGR sequences was deleted. As sRNAs themselves should keep certain lengths, and there might be some transcriptional units at either side, the sequences shorter than 120 bp were excluded, and the retained sequences were defined as IGRs (**Table 2).**

**Table 2:** The intergenic region (IGR) and sRNA information of KLDS 2.0603 and other 9 reference strains.

Sample Genome Name	Number of IGR	Average Length of IGR (bp)	Number of candidate sRNA
ATCC-15697	959	560	79
ATCC-15703	542	511	35
ATCC-25527	536	542	34
BB-12	518	695	27
KLDS 2.0603	514	512	24
Lactobacillus-acidophilus-NCFM	495	590	25
Lactobacillus-rhamnosus-GG	1062	529	110
NCC2705	595	503	39
PRL2010	712	507	53
UCC2003	672	566	70

### Prediction of promoters and terminators

Within the selected IGR sequences, the promoters were selected by Promoter 2.0 software (http://www.cbs.dtu.dk/services/Promoter/) [28], and terminators were selected by TransTermHP software (http://transterm.cbcb.umd.edu/) [29]. After getting rid of the duplicated sequences, the IGR sequences possessing complete transcriptional units were selected and the sequences containing tRNA and/or rRNA sequences were removed. Thus the sequences left were potential sRNA sequences.

### Quantification of transcriptomic data and identification of sRNAs

Quality Control (QC) analysis and trimming of the original transcriptomic data were performed by FASTX-Toolkit (http://hannonlab.cshl.edu/fastx_toolkit/) [30]. The low quality bases at each side of the reads were trimmed. In addition, only reads within which 80% bases had a quality of more than 20 and the lengths longer than 20 nt were kept. Those reads were called clean reads. The FASTQC software [31] (http://www.bioinformatics.babraham.ac.uk/projects/fastqc/) was utilized to exhibit the QC of reads and quality of reads after trimmed.

The clean reads were then mapped to the genome sequence by Bowtie software (http://bowtie-bio.sourceforge.net/) [32], and 2 or less mismatches were allowed. The information of the mapped reads was collected, whereas the duplicated reads caused by PCR were removed by Samtools software (http://samtools.sourceforge.net/) [33]. The sequences retained took part in the following analysis.

RPKM (Reads per kilo base per million) as an effective method of gene expression estimation was calculated as follows:

$$RPKM\;=\;\frac{total\;exon\;reads}{mapped\;reads\;\left(millions\right)\times exon\;length\;\left(Kb\right)}$$

The part of total exon reads/ mapped reads (millions) could be considered as the percentage of the reads that mapped to the gene, and was divided by gene length to get the percentage of total mapped reads within per unit length.

Gene expression levels was estimated by a combination of Cufflinks software (http://cufflinks.cbcb.umd.edu/) [34] and genome annotation files to obtain the differential expression genes of KLDS 2.0603 under different simulated GIT environments and to verify the predicted sRNAs accordingly. The high-confidence sRNAs were those IGRs with complete units of promoter and terminator, as well as with expression level 15% higher than the gene expression level at both sides.

According to the annotation information of KLDS 2.0603 genome and the promoter/ terminator prediction results of IGRs, the information of sRNAs such as location in genome and gene translation orientation was collected.

### The target gene prediction and construction of regulation network of sRNAs

The predicted target gene information of sRNAs was obtained by TargetRNA2 software (http://cs.wellesley.edu/∼btjaden/TargetRNA2/index.html). As regulating the expression of target genes was often the main function of sRNAs, the function analysis towards target genes could conduce to discover the mechanisms of sRNA regulation. Hence, sRNA regulation network was constructed through Cytoscape software (http://www.cytoscape.org/) to display the regulation function of different sRNAs straight forwardly [35].

### Gene expression profiling analysis

The informations of differentially expressed genes (DEGs) of KLDS 2.0603 after different simulated GIT environmental treatments were obtained according to the transcriptomic data. In order to search for genes that were significantly differential expressed with different treatments, the gene expression level was normalized by median method, and then the fold of gene expression level of treatments to control was also calculated. Values of |log2 Fold Change (FC)| > 1 and p < 0.05 were selected as the cutoff criteria for screening the DEGs.

## Results

### Survival of the different *Bifidobacterial* strains under simulated GIT conditions

**Fig 1:**
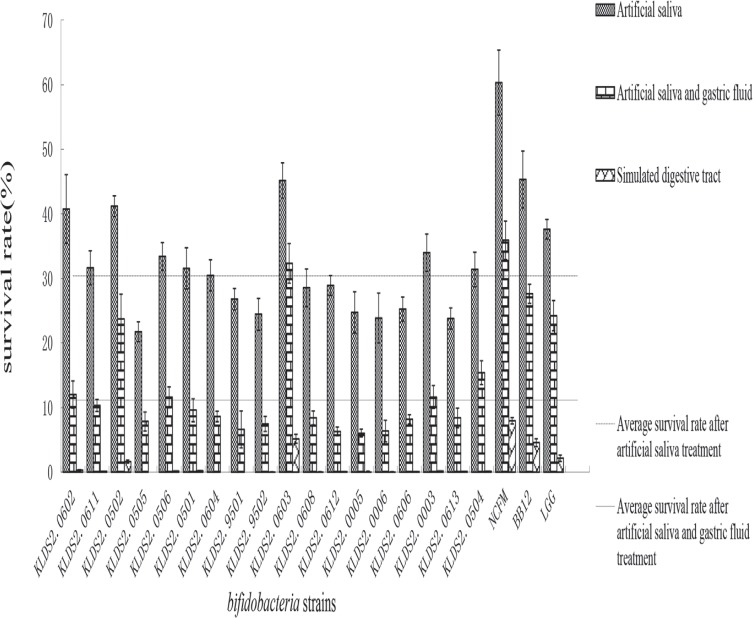
The survival rate of the *bifidobacterial* strains in simulated GIT treatments. The two horizontal lines indicate the average survival rate of the *bifidobacterial* strains after treatments. The upper line represents the average survival rate after artificial saliva treatment, and the lower one represents the average after artificial saliva and gastric juice treatment.

The survival rates of the 18 *bifidobacterial* strains and 3 reference strains were shown in **Fig. 1**, with significant differences among the survival rates of 18 *bifidobacterial* strains (*P* < 0.01). There were 9 strains with higher survival rates than the average (30.42%) after artificial saliva treatment in the 18 *bifidobacterial* strains, including KLDS 2.0603. The average survival rate of the 18 *bifidobacterial* strains after artificial saliva and gastric juice treatment was 11.16%, and 6 strains, including KLDS 2.0603 possessed higher survival rates than the average. However, the average survival rate of the 18 *bifidobacterial* strains decreased to 0.47% after artificial intestinal fluid treatment, and only two strains had higher survival rates than the average, including the KLDS 2.0502 (1.65%) and KLDS 2.0603 (5.15%). Among the 18 *bifidobacterial* strains, KLDS 2.0603 strain possessed the highest survival rate after sequential simulated GIT treatment. In addition, the survival rates of the 3 reference strains after sequential simulated GIT treatment were 7.96%, 4.57% and 2.17%, respectively.

### The adhesion analysis of the different *bifidobacterial* strains before and after simulated GIT treatment

**Fig 2:**
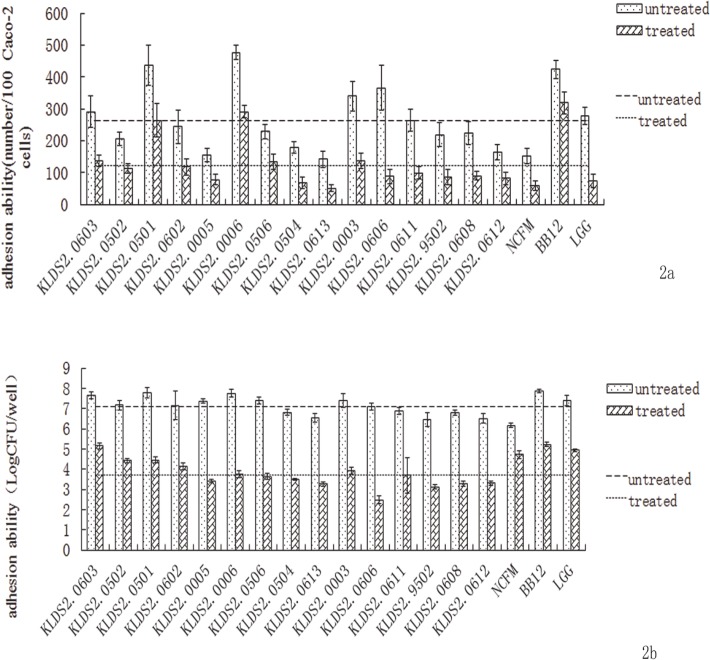
The adhesion ability of the *bifidobacterial* strains before and after simulated GIT treatments. **2A. Adhesion ability detected by direct microscopic examination method.** The upper line represents the average adhesion ability of untreated samples, and the lower line represents the average number of treated samples. **2B. Adhesion ability detected by plate count method.** The upper line represents the average adhesion ability of untreated samples, and the lower line represents the average number of treated samples. The adhesion ability is represented as the number of *bifidobacterial* cells adhered to 100 Caco-2 cells.

Based on the adhesion analysis of 15 *bifidobacterial* strains to Caco-2 cells, 3 strains didn’t retain survival after the **simulated GIT** treatment. The adhesion ability of the *bifodobacterial* strains before and after treatment was examined by two different methods (**Fig. 2**).

**Fig. 2A** indicated the adhesion ability of the *bifodobacterial* strains by direct microscopic examination method. The adhesion ability was significantly different among the 15 strains both before and after treatment (*P* < 0.01). An average of 262 bacterial cells of untreated *bifidobacterial* strains was adhered to 100 Caco-2 cells. There were 6 strains with adhesion cell numbers larger than the average, including KLDS 2.0603. The average adhesion cell number of the 15 strains after treatment decreased to 122 in 100 Caco-2 cells, and 5 strains including KLDS 2.0603, showed higher adhesion cell numbers than the average value.

Similar results of adhesion analysis were obtained by plate count method (**Fig. 2B**). Before treatment, the average adhesion cell number of the 15 strains was 7.11 LogCFU/well, lower than the other 8 strains including KLDS 2.0603. After treatment, the average value was 3.70 Log CFU/well, and KLDS 2.0603 was one of the 6 strains with higher adhesion cell number than the average.

### Comparative whole genome analysis of KLDS 2.0603 and other 9 reference genomes

As KLDS 2.0603 strain showed high survival rate and adhesion ability after simulated GIT fluids treatment, it was chosen as model strain to obtain whole genome analysis and transcriptome sequencing results compared with nine reference genomes. The determined genome sequence of KLDS 2.0603 strain consists of 1,946,899 base pairs, with a guanine-cytosine (G+C) content of 60.48%, nearly identical to genome sequence of *B. animalis* subsp. *lactis* BB12. Furthermore, the genome sequence of this strain contains 1,747 genes in total, including 1,678 coding genes, 52 tRNAs, 19 tRNAs synthetases as well as 5 rRNA operons, which also highly similar to that of BB12 strain (**Table 2**).

### Gene expression profile analysis in different GIT relevant conditions

In order to determine the sRNAs and genes involved in the resistance of KLDS 2.0603 towards GIT environmental stress, KLDS 2.0603 strains were treated with acid, bile salts and simulated GIT fluid, and KLDS 2.0603 strain without any treatment was as a control. Then the comparative transcriptome analyses of the KLDS 2.0603 strains in 3 different conditions and control were performed.

**Fig 3:**
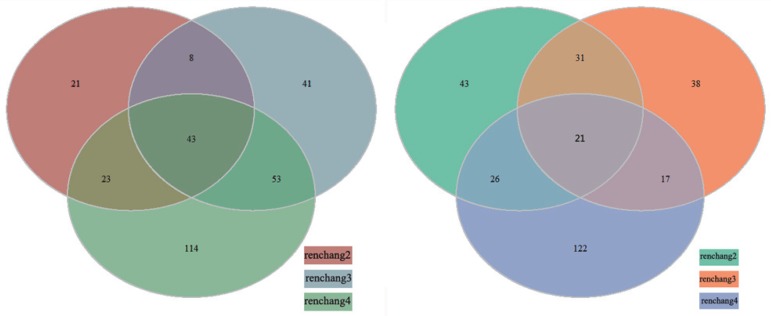
Venn diagram of the differential expression genes under acid, bile salts and the simulated GIT treatments. Picture on the left shows up-regulated genes, picture on the right shows down-regulated genes. renchange 2: acid treatment; renchange 3: bile salts treatment; renchange 4: simulated GIT treatment.

Quality-filtered reads were then mapped to the genome of KLDS 2.0603 and the mapped results were shown in **Table 2 in S1 File**. Afterwards, gene were annotated (**Table 3 in S1 File**) and gene expression profiling (**Fig. 3, Table 4 in S1 File**) of KLDS 2.0603 was also analyzed after different GIT environmental treatments. A total of 1656 genes out of 1747 genes (94.79%) were annotated in all 4 samples. The results showed that the number of differentially expressed genes of KLDS 2.0603 in simulated GIT condition was more than that in the other two treatments (acid and bile stress) compared with control, suggesting environmental stress in GIT was involved in a very complex multi-stress process compared with acid and bile stress. However, some genes were also up-regulated or down-regulated in the acid and bile treatments. In acid treatment, 38.84% up-regulated genes and 69.47% down-regulated genes were the same as in simulated GIT treatment. As to bile salts treatment, 35.51% up-regulated genes and 53.33% down-regulated genes were also the same as in simulated GIT treatment (**Fig. 3**).

### Expression level analysis of sRNAs in different GIT relevant conditions

The 4 sets of transcriptomic data were analyzed respectively, and 10 sRNAs in control and in bile salts treated samples, 11 sRNAs in acid treated samples and 9 sRNAs in simulated GIT treated samples were identified. The 9 sRNAs identified in simulated GIT treated samples were also predicted in the other stress treated samples. In the end, all of the 11 predicted sRNAs were selected as high-confidence sRNAs of KLDS 2.0603 and the genetic information was shown in Table 3 (Nucleic acid sequences in **S2 File**). Within the 11 predicted sRNAs, 9 sRNAs were located in the sense strand and 2 sRNAs in the antisense strand, indicating the diverse locations in the genome. In addition, the upstream genes or downstream genes of each sRNA undertake completely different functions, indicating that the 11 sRNAs might participate in various adjusting process in KLDS 2.0603.

**Table 3:** The genetic information of predicted sRNA in KLDS 2.0603.

sRNA name	Start site (bp)	End site (bp)	Strand	Length (bp)	Upstream Gene	Downstream Gene	Orientation
IGR-113	337, 052	337, 338	+	286	Signal recognition particle protein	cysteinyl-tRNA synthetase	→→←
IGR-130	406, 166	406, 479	+	313	Hypothetical protein Balac_0325	ribonucleoside-diphosphate reductase subunit beta	→→←
IGR-136	426, 611	426, 713	+	102	Response regulator of two-component system	Phosphate-binding transport protein of ABC transporter system	→→→
IGR-2	5, 224	5, 584	+	360	Hypothetical protein BLA_0004	DNA gyrase subunit B	→→→
IGR-217	707, 356	707, 488	+	132	Hypothetical protein	Nucleotidyltransferase/DNA polymerase involved in DNA repair	←→→
IGR-33	87, 444	87, 318	-	126	Integral membrane protein	N6-adenine-specific methylase	→←→
IGR-36	96, 352	96, 163	-	189	Serine/threonine-protein kinase PknB	Serine-threonine protein kinase	←←→
IGR-392	1, 506, 611	1, 506, 740	+	129	ATP-dependent helicase	Putative CRISPR-associated Csb2 family protein	←→←
IGR-466	1,779, 897	1, 780, 115	+	218	16s_rRNA	UDP-galactopyranose mutase	←→←
IGR-64	183, 658	183, 850	+	192	Amino acid permease	ABC transporter ATP-binding protein	→→→
IGR-93	286, 390	286, 859	+	469	phosphohydrolase	IclR-type transcriptional regulator	→→←
IGR-136	426, 611	426, 713	+	102	Response regulator of two-component system	Phosphate-binding transport protein of ABC transporter system	→→→
IGR-2	5, 224	5, 584	+	360	Hypothetical protein BLA_0004	DNA gyrase subunit B	→→→

The expression levels of the 11 sRNAs in the three treatments were also determined (**Table 4**). The acid treatment induced a significantly high expression of IGR-392, which was 4.12 fold than that in control. The bile salts treatment, on the other hand, leaded to higher expression of all the sRNAs except for IGR-2. As to simulated GIT treatment, 8 of 11 predicted sRNAs were down-expressed, except for IGR-2, IGR-130 and IGR-36. The results of up- or down-expression for sRNAs of KLDS 2.0603 in simulated GIT conditions suggested some sRNAs might play a vital role in resistance to environmental stress at transcription and post-transcription level.

**Table 4:** The sRNA expression levels of KLDS 2.0603 under different simulated GIT environment treatments.

sRNA	Acid treatment/ Control	Bile salts treatment/ Control	Simulated GIT treatment/ Control
IGR-113	1.63	3.86	0.29
IGR-130	1.26	1.25	0.94
IGR-136	0.98	1.84	0.59
IGR-2	0.70	1.05	1.25
IGR-217	2.15	3.26	0.33
IGR-33	0.90	1.44	0.26
IGR-36	1.11	1.24	0.86
IGR-392	4.12	1.55	0.65
IGR-466	0.96	1.99	0.47
IGR-64	1.92	1.76	0.50
IGR-93	2.50	1.19	0.63

### Functional categorization of differentially expressed genes regulated by sRNA in the three treatments

**Fig 4:**
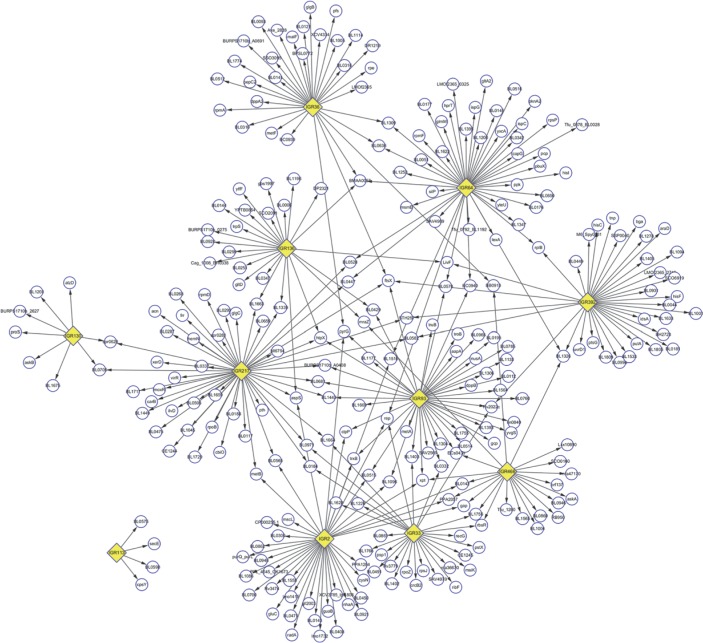
The sRNA regulation network of KLDS 2.0603 strain. The sRNA regulation network was constructed by Cytoscape. The yellow rhombuses represent sRNAs, the circles pointed by arrows represent the target genes. As many hypothetical proteins (like the one labeled by BAD_1128) in KLDS 2.0603 genome have homologous genes in ATCC-15703 genome, the hypothetical genes were represented by the homologous genes in this network.

**Fig 5:**
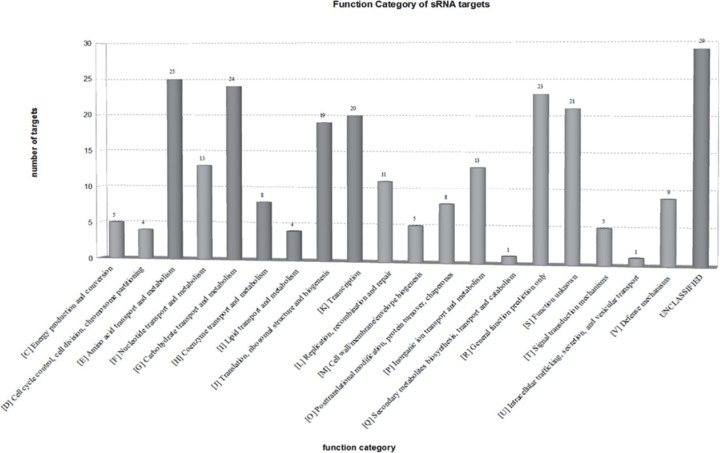
Function categories for the target genes of all 11 sRNAs. The COG category of each target gene was identified by BLAST tools and COG database. Then the target genes with similar COG categories were gathered to form the graph. The numbers on each column represent how many target genes are within the categories.

To better understand the role of sRNAs in different stress conditions, sRNA-regulating target genes were predicted using Targe tRNA2, taking ATCC 15703 as reference genome. The number of the target genes regulated by each sRNA was shown in **Table 5 in S1 File**. Then the sRNA regulation network was constructed by Cytoscape software. These results showed that 276 target genes were regulated by these 11 sRNAs, among which 48 were co-regulated by at least 2 sRNAs. In particular, genes such as pyrG, htpX, aspS were co-regulated by 3 sRNAs and the gene ftsX was co-regulated by 4 sRNAs (Fig. 4). The Clusters of Orthologous Genes (COG) analysis of target genes regulated by sRNAs was performed using COG category and BLAST data from NCBI, and 248 of 276 target genes possess COG information. The COG categories data suggested that target genes regulated by sRNAs were mainly involved in amino acid and carbohydrate transport and metabolism, translation ribosomal structure and biogenesis, as well as transcription (**Fig. 5)**.

## Discussion

In this study, we identified the homology between the selected KLDS 2.0603 strain and BB-12 by the comparison of KLDS 2.0603 genomics data with other 9 reference strains. In addition, the transcriptomic analysis of KLDS 2.0603 strain under simulated GIT environment treatments allowed us to identify 11 sRNAs and 276 target genes involved in the response mechanism against environmental stress. The COG information obtained from 248 out of 276 target genes indicated that the regulation of the 11 sRNAs was involved in amino acid and carbohydrate transport and metabolism, translation, ribosomal structure and biogenesis, transcription, and cell wall/membrane/envelope biogenesis.

The results showed that differentially expressed genes of KLDS 2.0603 under different stress conditions were not always homogeneous. Thus it seemed that the regulation mechanism of sRNAs was different under different treatments. This study also indicated that all the sRNAs up-regulated except for IGR-2 in bile salts treatment, while in simulated GIT treatment, IGR-2, IGR-130 and IGR-36 up-regulated and all rest sRNA down-regulated. COG analysis indicated that the target genes regulated by IGR-2 sRNA mainly took part in amino acid and carbohydrate transport and metabolism, ribosomal structure and biogenesis (unpublished data). Its adverse expression in different stress treatments might be due to the different pathway ay because IGR-2 regulates gene expression through involving in. In addition, the influence of simulated GIT treatment on KLDS 2.0603 strain was not merely the acid stress plus the bile salts stress. The mechanism involved in resistance of *bifidobacteria* to GIT condition was very complex and the genes involved were likely to response to other stress, such as digestive enzyme and salts.

The sRNA regulation network showed certain important target genes were regulated by at least 2 sRNAs, such as *pyrG, htpX* and *aspS* co-regulated by 3 sRNAs and *ftsX* co-regulated by 4 sRNAs. The *pyrG* gene encodes orotidine 5’-phosphate decarboxylase, which catalyzes the conversion of orotidine monophosphate to uridine monophosphate to maintain a low intracellular uracil concentration [36]. As a cytoplasmic membrane-bound Zn^2+^ metalloprotease, the *htpX* gene was involved in membrane protein quality control that was important for growth and survival of the cells, especially under stress environment [37]. The *aspS* gene encodes Aspartyl-tRNA synthetase that played an crucial role in the accurate interaction of an amino acid with its cognate tRNA, and thus it was important for protein synthesis [38]. The *ftsX* gene encodes a transmembrane domain of a putative ATP-binding cassette transporter which was related to various biological process, including EPS biosynthesis [39], carbohydrate uptake and metabolism systems [40], response to acid [41], resistance to bile salts [42], etc. All the four genes played potentially crucial functions in the survival of the KLDS 2.0603 strain under stress condition.

In particular, it was found that the sRNA named IGR-113 regulated all its target genes only by itself, which was different from all other co-regulating sRNAs. The sRNA named IGR-113 was predicted to regulate 4 target genes, including *secE* and *cpsY*. The *secE* gene encodes a V-shaped protein that enwrapped the protein-conducting pore secY, and thus played a supporting role in protein translocation [43]; while the *cpsY* gene was a transcriptional regulator that regulated methionine biosynthesis and uptake pathways [44]. As the target genes of IGR-113 performed important functions in cell survival and cell adhesion, IGR-113 should have taken part in the resistance of KLDS 2.0603 towards environment stress, which was confirmed by the regulated expression level of IGR-113 under simulated GIT environment treatments.

Owing to the complexity of post-transcriptional regulation of gene expression, the genes or regulators related to the resistance mechanism might be ignored in this research on sRNAs. In addition, as the real GIT environment was more complex than the simulated conditions, the genes taking part in the resistance under simulated conditions might not be consistent with genes in the real conditions. Moreover, this research focused on the regulators and genes regulated by them involved in stress response, but with no direct evidence for the specific mechanism. Thus, further researches with a combination of transcriptomics, proteomics as well as metabolomics analysis of the KLDS 2.0603 strain under GIT environment conditions should be performed. In conclusion, our findings derived from the functional genomics and transcriptomics analysis of KLDS 2.0603 provided a relatively complete and thorough analysis of resistance mechanisms at transcription and post-transcription level towards environmental stress for the first time.

## Supporting Information

S1 File Table 1 Bacterial strains used in this research; Table 2 NA-Seq data quality control (QC) and mapping results of KLDS 2.0603 strain; Table 3 The expression gene number obtained from KLDS 2.0603 transcriptomic data after simulated GIT condition; Table 4 The differentially expressed gene number obtained from KLDS 2.0603 transcriptomic data after simulated GIT environment treatments; Table 5 The number of target genes predicted for each sRNA. (DOCX)

S2 File Nucleic acid sequences of the 11 predicted sRNAs. (DOCX)
