# Supplementary material for: Genome-Wide Identification of Small RNAs in Bifidobacterium animalis subsp. lactis KLDS 2.0603 and Their Regulation Role in the Adaption to Gastrointestinal Environment
Source: PLoS One. 2015 Feb 23;10(2):e0117373. doi: 10.1371/journal.pone.0117373 (PMC4338058; doi:10.1371/journal.pone.0117373)
Supplement: S1 File — (DOCX) [file pone.0117373.s001.docx]

**Table S1 Bacterial strains used in this research**

| Species | Strain | Source |
| --- | --- | --- |
| *Bifidobacterium adolescentis* | KLDS2.0005 | KLDS-DICC |
|  | KLDS2.0506 | KLDS-DICC |
|  | KLDS2.0612 | KLDS-DICC |
|  | KLDS2.0003 | KLDS-DICC |
| *Biofidobacterium bifidum* | KLDS2.0006 | KLDS-DICC |
|  | KLDS2.0502 | KLDS-DICC |
|  | KLDS2.0602 | KLDS-DICC |
|  | KLDS2.0606 | KLDS-DICC |
|  | KLDS2.0613 | KLDS-DICC |
|  | KLDS2.9502 | KLDS-DICC |
| *Bifidobacterium lactis* | KLDS2.0501 | KLDS-DICC |
| *Bifidobacterium longum* | KLDS2.0504 | KLDS-DICC |
|  | KLDS2.0608 | KLDS-DICC |
|  | KLDS2.9501 | KLDS-DICC |
| *Bifidobacterium infantis* | KLDS2.0505 | KLDS-DICC |
|  | KLDS2.0604 | KLDS-DICC |
|  | KLDS2.0611 | KLDS-DICC |
| *Bifidobacterium animalis* | KLDS2.0603 | KLDS-DICC |
|  | BB-12 | KLDS-DICC |
| *Lactobacillus rhamnosus* | GG | KLDS-DICC |
| *Lactobacillus acidophilus* | NCFM | KLDS-DICC |

KLDS, Key Laboratory of Dairy Science (China)

**Table S2 NA-Seq data quality control (QC) and mapping results of KLDS 2.0603 strains**

| Sample | Raw reads number | Reads number after QC | Mapped reads number | Mapped reads number without duplicate |
| --- | --- | --- | --- | --- |
| Control | 53,558,892 | 47,668,948 | 39,621,883 | 724,912 |
| Acid treatment | 59,137,344 | 52,315,339 | 41,923,616 | 763,541 |
| Bile salts treatment | 59,321,866 | 52,567,989 | 42,609,820 | 619,782 |
| Simulated GIT treatment | 69,065,028 | 61,354,676 | 27,438,087 | 672,172 |

**Table S3 The expression gene number obtained from KLDS 2.0603 transcriptomic data after simulated GIT condition**

| Sample | Total gene number | Expression gene number  (mapped reads number ) | IGR number |
| --- | --- | --- | --- |
| Control | 1747 | 1700 (97.3%) | 24 |
| Acid | 1747 | 1691 (96.79%) | 24 |
| Bile salts | 1747 | 1689 (96.68%) | 24 |
| Simulated digestive fluids | 1747 | 1727 (98.85%) | 24 |

**Table S4 The differentially expressed gene number obtained from KLDS 2.0603 transcriptomic data after simulated GIT environment treatments**

| Samples (compared to control) | Up-expressed gene number | Down-expressed gene number |
| --- | --- | --- |
| Acid treatment | 121 | 95 |
| Bile salts treatment | 107 | 145 |
| Simulated GIT treatment | 186 | 233 |

**Table S5 The number of target genes predicted for each sRNA**

| sRNA | Target Number |
| --- | --- |
| IGR-113 | 4 |
| IGR-130 | 8 |
| IGR-136 | 24 |
| IGR-2 | 41 |
| IGR-217 | 51 |
| IGR-33 | 27 |
| IGR-36 | 31 |
| IGR-392 | 35 |
| IGR-466 | 21 |
| IGR-64 | 47 |
| IGR-93 | 42 |
