# Supplementary material for: Genome-Wide Identification of Small RNAs in Bifidobacterium animalis subsp. lactis KLDS 2.0603 and Their Regulation Role in the Adaption to Gastrointestinal Environment
Source: PLoS One. 2015 Feb 23;10(2):e0117373. doi: 10.1371/journal.pone.0117373 (PMC4338058; doi:10.1371/journal.pone.0117373)
Supplement: S2 File — (DOCX) [file pone.0117373.s002.docx]

**Nucleic acid sequences of the 11 predicted sRNAs**

**IGR-113**

GTTCTTCACCAGGTTTTCCTCTGCGTAATTGGGGACAGATAGCGGGTCTATCTGTCCCTTTTTACGTCCCTTCTTGAGTTCATCGTGGCGCATTTGAATTGAGGTATCGCATAAGGCAACCATTGGCGGCTTCGCTGGTTGGGGTGAGGTTGGGATTGCAATGGCGACACGTGTAGCTTCCATATGGTGGTCGTCGTGAGATTGACGCTGCAAGTGTGGCATGCGGATCTGTCTGTAGTAGTCGTCGCGAGATTGAATTTGCAAGTGTGGCACGTCTGCGGGTCTCTTGGGTTGTCTGTTTTGCATTCCGAATCTCAGTAAGGTTCGTTGAGGCGCGCTCGGTGGTTTGGGTGAGGCTCGGCGTGCAAAGGTGGCGCGTGAATCTGTGCTGCGATGGTCGGTGTGAGGTCGAATCTGCAAACATGGCATGTTAGATGTCGCTTACTGGCTGGGTTGAGATTCGAGTTACAAACACGACATGTTTGCGAAGGGCCGAACATGCCGATCTGAGACCGCAATTGCAAACGCGACACGTGCAATGTCAAAAGTAAGACGACGTAAATAGGAATAGACAGAAAGATGTATCCGCCTCAATCTCCATGTGAAGATGGGCGGGCAGAGAAGAGAGAAATTTAGTATAAATAAGGGTGATGTTAGCGTAAAAAGAGATAGATGGAAGCGCGCAATGACCCTTTTTACTCAGGGAATGGGGCATGGGAGGGCATAGGGAATGGGTGCGTGGAGATTGGATGTAAATGGGGACAGATTGGCTCGCTATCTGTCCTTTTTTATGCAGAAATAAGATGCATGAGATGGCATCGGTATTAGCGTAAAAGGAGACATCTATAGGCTTCCTGCTGTCCCTTTTTGCAAAGAAGGCAAAGAAGTCCGCGCCCCCGGTGGGACTCGAACCCACACTGGGTGATCTGTCCCTTTTTACGCAGGAAATAGAACGCTGAATTTTTATACCGGGGATAGAGCGCTGAATTGGACGTAAATGGGGACAGTTAGAACCGCTATCTGTCCCCATTTACGCAAAGAAGTCCCTTTCGCAAAGGAACTT

**IGR-130**

TGACGTAGGAGCTGCCGGAACCCGAGAAGAAGTCGTGGTTCTCGTCGGCATTCGGCGAGAGCGCGGCCAGGATCGCCGGGTTCACGTCGCAGATCTCGCTCGGGAACAGGGCCGGGTAGCCGAGGTTCATCAACGCCTTGTTCGCGTTGTAGTGCAGGAACTTCTCCACGTCGTCGGACAACCCTACCTCGTCGTAGAGCGAGTGCGTGTACTCGACCTCGTTCTCGTACAGCTCGTTCAGCAGATCGTAGGTGTAGGTCTCCATCTCAGCGCGCTTGGCGTCGCTCGCCATCTCGAGGCCTTTCTGGTATTTGTAGCCGATGTAGTAGCCATGCACGGCCTCGTCGCGAATGATCAGTCGGATCACGTCGGCCGTGTTCGTCAGCTTCGCATGGCTCGAGAAGTACATGGGCAGGTAGAAGCCGGAGTAGAACAGGAACGATTCGAGCAGCGTCGACGCCACCTTGCGCTTCAGCGGGTCGTCTCCCTCGTAGTAGTCGAGCACAATCTGCGCCTTGCGCTGCAGGTGCGGGTTCTTTTCGCTCCAGTCGAAGGCCTCGTCGATCTCGCGTGTGGAGCACAGCGTGGAGAAGATCGACGAATAGCTCTTCGCATGCACGCTCTCCATGAACGCGATGTTCGTGTACACGGCCTCCTCGTGCGGGGTGAGCGCGTCGGGAATCAGGCTCACGGCGCCCACAGTGCCCTGGATCGTGTCGAGCAGCGTGAGCCCGGTGAACACGCGCATCGTGAGCGTGTGCTCGGCGTCGCTCATCTGCCGCCAATCCGGAATGTCATTCGAAACCGGCACTTTCTCGGGCAGCCAGAAATTGCCGGTGAGACGATCCCAGACCTCCAGGTCCTTCTCGTCATCCAATCGGTTCCAGTTGATGGCGGTAACGCGGTCGATGAGTTTTGGCA

**IGR-136**

CCCATTGCTCTACCAGCTCACCCGTGAGGGGTACGAGGCCCATGGAGCCGCCACCGGGGAGGAGGCGTGGGAGCTGTATGAGGAGATGGAGTTCGACCTCGTGTTGCTCGACCTCATGCTGCCCGGCATCTCAGGCACCACACTCTGCAGGCGCATGCACGAGCTGCGCCGTGTGCCGATCATCATGGTGACAGCGAAATCAACCGAGAATGATGTGATCGTAGGGCTCGAAATCGGTGCCGACGACTATGTGACCAAACCGTACTCCTTCCGCGAACTGCTCGCCAGGATCCATGCGGTGTTGCGTCGCAGTCTCGAGCCACGGGCTTCTGGAACACCCGGAGGGAACGATGAGACGCTTACATGCGGGCCCATCGAGATGTCGGTGGCCTCGCACGAAGTGCGCATTCACGGTGAACTGACCCAGTTCCCGCTCAAGGAATTCGAGGTGCTCGAATACCTGATGCGCAATAAGGGCCGCGTGCTCACCAGACAGCAGCTGATCGGCCGCGTCTGGGGCAGCGATTACGTGGGTGACACGAAAACGCTCGACGTGCATGTCAAGCGCATCCGGGCGAAGATCGAGGCCGATCCGTCGAATCCGGCACTGCTGACGACGGTGCGCGGTCTCGGGTACAGAATCTGCGACGAACGTTCACGCTAAATTCACCGTTCGCCTCAATACGACTCCCCACCCTCCTCTACACTGA

**IGR-2**

GGCATTGCCGGCCGAGGAGTTCGAACATGTCTCGGCACGCGCCGGACTCATTGCGGAACGGCGCGTGAAGCGCGAGGAGGCGCTCGAGAACTTCGGCAAACCGGGGCGCGACGAGGTGGCGTTCGGCTCGTTGTTCGCGGGGATCGCCAAACGGGCCGGCTGGGTGCCCCATATGAAACTGGCCCAATTGCGTCACGATTGGGCGTCGATCGTGGGCGATGTGATCGCACGCAACACCTGGGTGGGCGCCATCGACAACGGTGTGCTCACCATCCACGCGAAGTCGCCGTCGTGGACCACGCAGCTCACGTTCATGCTGCCCGAGCTGCGCGAGAAGGTGCTCGAGCGCCTCAATGGCCTCGACATCCGCGACATTCGCGTCACAGGCCCGCAGGCGCATGGGCCCGCCACGCGTCGCAAGCAATATGTGCGGCAGAACATGCACTACCGTCCACAGTGATCCAACTCGGACCGCTTGTGCGGCGAAAAACCAGGAAATCCGCTACAAGCGCGCTAAGAACACGAGTCCCCCCATGCGAGTAGAATCCTTAGTAGTGTAGTTGAACGCCCACAAGGGCGTTTTATTCGTGTTACAGGGCATATCGCCTGT

**IGR-217**

ATGCGTGGCGAGGGGCCTGGATCCGCTTGACGCCAATCTGGTGGTGGCGGATGTGTCCCGTACGTCGAAGACCATATGCCTTGCGGTGTCGCCGGCGCTGAAATCGTTCGGGTTGGGGTCGCGCGCCAGACTGTTCGAAGTGGAGGAGCGTGTCGGGGTGCTCAACGAGCAGCGGCGTCTGCGTGCGCCGGGGCACAGGCTTGCGGGGTCGTCGGTGAGCGCGGCGCAACTGCAGGCTGATCCACGGCTCGGCATTGAATATATTGCCGCCAAACCGCGCATGTCATACTATTTGGACACCAGTGCGAAAATCTACGGCATCTATTTGAACTACGCCTCGAAAGAGGATATCCACGTGTATTCGGTGGACGAGGTGTTCATCGATGTCACGAAATATCTCAAGTATTTCGGGTGCTCGGCCCATGAACTGGCACGTCGCATCGTGGCCGACATTCTCGAGCAGACGGGCATCACGGCCACGGCAGGCATCGGCGCGAACCTGTATTTGGCGAAAGTGGCGATGGATATCGTTGCTAAGCACATCCCCGCGGATGAGGACGGCGTGCGCATTGCCGAACTCGATGAACGCAGGTATCGCGAACTGCTGTGGTCCCACCGGCCTCTCACCGATTTCTGGAGGGTGGGCCGTGGCATAGCCCGCAAATTGGAGCGGCAGGGAATCATGACCATGGGAGACATTGCACGGTGTTCGTTGGGAAGGGCCGGCGAATATTACAACGAGGATCTGCTCTACAGGTTCTTCGGCGTGAATGCGGAAACGCTCATCGACCATGCTTGGGGTTGGGAGCCATGCACAATCGCCGATATTAAGGCATATACGCCGGAGAATTCCAGCCTGAGCATAGGTCAGGTGCTTGCGCGTCCCTATCCGCATACACAGGCCCACACCGTAGCGAGTGAGATGGCGGACTGCTTGGCGCAGGATCTCGCTGCCAAGGGGGCGGTGACGGACCAGGTCGTGCTGACAGTGGGATTCGACAGGGAGAGTCTTATGGACGGCCGGTACCTCGGGCCCGTAGGCAAGGATCACTATGGGAGACCAGTGCCCAAAAGCGTTCATGGCTCATGTGATCTGGGGCGGTTCACGTCATCGGCCACTGCCATTCGACAGGCGGTGGTGGATGTTTTCGAACGCATTGCGGATTCGCGGCTGTATGTCAGACGCCTCACGGTTGTAGCCGGCCGGGTTCGTATGCTGGGGGAGACCCAGACCTTCGAGCAGCCGAGTCTGTTCGAGGATCTGGAGGGCTCGCAAACACAGCAGCAGGCTACGGCAGATCGCAAGGAACTGAGTCTGCAGCGCACCGTCAACGCACTGCGCAGCCGGTTCGGCGCGGCATCGGTGATCAAAGGCGTGAACCTCGATGAGGGGGCGA

**IGR-33**

GTTCCTCACCCGCGTTTACATGGCACTGCGATGGCACGGTGTGCGGATGCTTCCGTATGCCGTGCCATTTCTGTAGATTCAGCGGTCGAACTTTCGGAAGGTTCCGCGAAGCAGGCCATTTCAGGGCATCTTTCCGGCAGATTGTTCGGAAGGTTCCGTGAGATGTGCCGTCGCGGGGTGCATTCGCGGCGTATCCTTCGGATGCTTCCGCAGATTGAGCCATTCAGATGCGAATCTGTGGCATATTGTACGGAGTCTTCCGTGTGATATGCCGTTCGGCGCTCGCGGGTGTCGGTGCGGCTGGTCGGCTGTGCCAATCTGGTCTCAGATTGGCACAACATACGCGCAATACCGCGTGACCTTTGCAAAGCGAGTCCCGTAATGACAATGAAGCGGCTCGGCTACAGGGCCGTATTTGCACGGGCAATCTCATATTGGTGAGGAGGCGGGAGACAACATTGCCTCACACGGCATTTTATTAGGTAATCGACGTAAATATGTCGTGTGAGAAGAAGGGGTGAGACTGCGCAGTGCTCGACGCGCCATTGCACATGCGCGGATCTACAGGTACCGCGGCGCCGCGGTCTGGCAACGGCGGGCAAACAGGTGTTCGATTGCGGTGCCGTAATCTGCGGAGTAGGGAAGTATGAATTCGAACATGTGTTTGAGTTACACGCATGTAGTTTTCCACAAATGTGGAAAACGTTGTGGATAACATGGGTATAGCGTATGGATAAGTGCTCAAGTTATCCACATGGTTATGCACAATCGGTGCAATTCCAATGGTTTCTGCTGTGGAATATTGTGGACAACGTTATCCACAATGGGCAGCGGGGAGTGTGGGCAATCTAGAAATAGGCAACAAGCTGCACGGTATGCCCGTATATAGTGGCAAAATAAGGCAATTTCGGCATGTT

**IGR-36**

GGAATCGGTACCGGCATCGCGCTGCGTCTGCTGCGCGAACGGGAAGGCGGATTGATACGTTTGCAGGTCATGGTGCGGTGCACTCGTGACCCGGCGGGGGACTCGTCCGTTGATGTTCACCATGGTGGTGTCGCTCAGCGCGGTCTGCTGGTCGAGCAGACGGCGCTCGATGCGGGAGAGCACACGCGCCACGGTCAGCGCGTCCTGCGGACGATCCGCAGGGTCTTTCGCAAGCATCGACATGACGAATTGGGAAAGCTGGAAATCCACGGTGTTCGGCAACGGGGGCACCGGATTGTTCACATGGGCGGCGGCGATGTCGACCGGCGTCGCCCCGGTGAACGGACGATGCCCGCACAGGCCCTCGTAGGCCACGACACCGAGCGAATAGATGTCGGACTGTGGCGTGGCCTGCTGGCCTTGCGCCTGCTCCGGCGAGATGTACTGCGCGGTGCCCACCACCATGCCGTCCTGCGTGATCTGCTCCTGGTTCGTGGAGTAGGAGACGCCGAAATCGGTGATTTTCACTTCGCCGTTGTCGGAGACCATGATGTTCGCCGGCTTCACATCGCGGTGGATCACACCATGCGAGTGGGCGACGAACAGCCCGCGGGCGGTCTGGATGAGAATAGGCAGCAGACGAATCGGGTCCATGGGGCCATCAAGCTCGTGGTAGAGGTCTGCCAACGACTTGCTCGGCACATATTCCATGATGAGGAAGCCGATGCCGTCGTGCTCGTAGTATTCGAACAACGCGGCGATGTTCGGGTGCGCCAGATTGGCGGAATTGCGGGCCTCCGCACGCAGGCGGCGCAGTTTGGACTCCTGGTTCGTCGTGTCTTTGCGCAGCGCCTTGATGGCCACGGGACGACCGAGCTGAATGTCGTAGCCCTTCCACACCTCGCCCATACCGCCCTGGGCCAGACGCGAGTCG

**IGR-392**

CCATAGGAATGAGTAAGCCCCCGCCAAAGTGCCTGCTCTGCCCGATAGCGAGAATCTCATCTTTAATCACATTCTCAAATGCAATCAATCCGGTCGATGCCGTAATCATGAAGCCCTCACGCATTCTGTGCACATAATCCCCCATATCTGGGCGAGCCACGGTACGCGCTGCAAGAATACGTACAAAAGAATCTCCTGCAGTCACTGCATCAATGAGATCCCAGTACTTACCCCTGCCTCTTTTTTCGGCAATTTGCCCACGGAACACGTTCCGAAATACGTGCCCTATGGAAACCGCAAGATTGCATTCCGCGGTCCACGACCTGCTTTGTCCGGCGGGGCTTTTTTCAGCTTGTGTTTCATTGACCGAAAATGGCATAGGTGACCATATTCGATGCATGCCATCCCTTGGTTTACTCCACAGATGCACCCCTTCTCCAGGGATTGGATTCCCTAATCGGAGTGCAGGCTTGCTGTGTGAATAGTATATTCTGGAGTTGGCCAACGAACGTACTAGCGTACCCAATTTCCGCAATTCCCCAGCATCCATTTCGCTGGGAATCATCAAAATGAAGGCAGGAAGAGACTCTGCGATTCTCTGGTCTGCAATCAACGACGCATAATCTTTCCGCAGAACTTGAATGGCGATGTTATTCGCCGGACGTTTAACCATAGAATCGGTGGACCACCGACCGGTCAGATATGGACTAACATCCGTACCCCAACGTCGCACTAATAAGCGGTGCAACGCCACACACCATGCAGTCAATTCATCATCCCGAGGATTCCACGCCGAAGCATTCGACTCCACGCGCGCGGGAATGATAATCATACGAGTCCATGGCAGCTGTACTGCCGACTTGGCTTGTTTCTGAGGGCTGTAGGTACTGCGATGTACACACTGCAATCTTTTCTCAGATCTGACTTCTGTGTCTTTTTTTGTGGATGAATCCTTCGGAATCTTAGAGGCTGGATGTGGATTAGCCTGAGTGTAATGCTCCATCAGCTCCTGATGAAGCCCCGGCGCAGGGCAAGGCAATTCGCGCGCACGCTCGACTTCCATCAATGATTGTGATTCCGGACGCAGCATATACGAATCAGGAAGGGGGAAATCAAATCCCTCCTCGACAGTAATACGTACAGGACTTCCAGCGCCACCCAAGAATGGAATCTCCCAACACAAATCTGCAATTGTACTGCACTCTTCGTCGTCTGGGCCATTTTTCCACTCGAGGATCATATCCCCTTGTTCATCAGTACGGTACATCACAGCCGACCGGGCCCGCTCCGCTTTGGGAGCTTTGTCTGCTTTACGCCTATAAGCAATGGCATTATGGGAGGTTGGAGATTGCGAAACTATCTCCGGAAAACGAATCGCTTCCGGCGGATTCGATTCCAACCATTCGAGTGCTGCGTTCAAACGCTTGTCAAGCATACCCTGTGAATTTTCAGATGAAAAAACGGTATGTGCGGCTGATACCATTGCCTGATATAAACGCATGGGAGTTGGAAAAGATTCCTTCTCCCCATATTCGCTTGCTCCTTGATAGGAAGCAAGAAGGAAGT

**IGR-466**

TCCATTTCAAAAGAAATCAGACGGACCCCCTAATAAGGAAAGGGAGCCACACAAAAAACCTCCGCCATCCATCAAAACCCGACAGGATTACCCACACGAAACCATGCAGGCGGACAACGGAACTGGCATCATAAAAGACGACCAAGAACCACAACGGCCACGACAGCCGAAGCGACATCAACCTTGGTCAAGTAGTACAGTACACGCTATTGAGTTCTCAAACCACCACCACACACCCACAACCCCGACCCGGGAACCCCGGCAGGCCGTGAGCGGCAGCAAAAGAAAAACCTACACCACAACCCCCACCCACGCAACCCCACCCCCAACCACACACCCCCAAACCCCAACAAACACAACACCACCACCGGCGTGTCGAAAACCAGCCAAAACACCAACCAACACCCACACACAAAAACACCAACCACACCACACAGACGAAACCCACCACGAGCGCAACAACGCAGAACGGGAACCCCGAATAGGGGTTCCCGTCCGCATTCAGAAAGCG

**IGR-64**

TTCCGTATGGCATGCCGTTTTCAGACTTCTTCGCGGTCGGTTTTGCGGATGCTTCCGAAAGCTATGCTGTTTTCAGACCATTCCGTGGCGCGTTTCTCGGATACCTCCGAGAGGCATGCCATTTTGGAGCCTTCTGGCAGTACGTTTCTCGGATGCTTCCGTACGCTGTGCTGCGGGGAAGACGGCCTGCGGTTTGGTTCTCGGATGCTTCCGTGAGATGTGCCTCTGATGGGGTGGTTTCTGGCCCATCTCTCGGATGCGTCCGTATGCTGTGCCGCTGCAGGGGTGGTTTGCGGTCCGTTGTGCGGATGCTTCCGTAGGCTGTGCCATTTTAGATTCCTTTGTGGCACGTTTCTCGGATGCTTCCGAAAGCTGTGCCGTTGGCGGGGTGGTTAGTGGCACATCCCACGGATGCTTCCGTATGTTGCACCGCATTGGCGGGCAGCACGCGGGGGCGGCCACTGTACTCCAGATTGGTTTTCGCATAATCTATATATGTAT

**IGR-93**

TGGTGTGACGATGGCTCGGTGGCCTCGATTTGCAAAACGTTGGAATTCCAACATTGTTATGGTCATGGGTTTGTGGGTGCCGTCACACCAAAGCGGTTTGCGTCACACCAAAACCATGTGAGCGTGGGAGACGTTGCCGGCACGACGTTTGGCTCGTCTTGCCGTCGTCTCGTTGTGTGTTACGGCCTTTTGGCTTACGGGCTGGGATTTTCTGTTACGGGCTAAGGTAGCCGCTACGGGCCAAGATAACGCTTACGGGCTAGGATTTTTGGTTACGGGCTAAAAGGGGCGTTGTATTCGGCAACAACAATCGTTGGAAAATGGCGGTTTTGTGTTTTTGGGGAATTGCTGGATACTTGCCAGGTTGCAGATTTTCGCTAGCCCGTAACCGAAAGGCCTAGCCCGTAAGGAGTTGGCTGGCCCGTAAGGGGCCCCATAGCCCGTAACAGCTGGTGACATCCCGTAACGGATAAGGACATCCCGTAAGAGATTATGACATCCCGTAACAGAAAACCTTAGCCCGTAACGTTGTATGAGACGGAGACGCGAAAAGAGAAAGGCGGCCAACCGTGGGGCTGGCCGCCTACTTGCGGGGTTGCGGAATGTATGTAGGTCGGTTGCCTACTTATGGGGTTGCGGAATGTGCGTAGGTTAGTTGCCTACTTGAACGCGATGATATCGGATG
